# DNA Cytometry and Nuclear Morphometry in Ovarian Benign, Borderline and Malignant Tumors

**DOI:** 10.3889/oamjms.2015.104

**Published:** 2015-10-01

**Authors:** Amina A. Gamal el Din, Manal A. Badawi, Shereen E. Abdel Aal, Nihad A. Ibrahim, Fatma A. Morsy, Nermeen M. Shaffie

**Affiliations:** 1 *Pathology Department, National Research Centre, Cairo, Egypt*; 2 *Community Medicine Research Department, National Research Centre, Cairo, Egypt*

**Keywords:** Ovarian, serous, mucinous, borderline, DNA ploidy, nuclear area

## Abstract

**BACKDROUND:**

Ovarian carcinoma is a leading cause of death in gynecological malignancy. Ovarian surface epithelial serous and mucinous tumours are classified as benign, borderline, and malignant. The identification of borderline tumours most likely to act aggressively remains an important clinical issue.

**AIM:**

This work aimed to study DNA ploidy and nuclear area in ovarian serous and mucinous; benign, borderline and malignant tumours.

**MATERIAL AND METHODS:**

This study included forty ovarian (23 serous and 17 mucinous) tumours. Paraffin blocks were sectioned; stained with haematoxylin and eosin for histopathologic and morphometric studies and with blue feulgen for DNA analysis.

**RESULTS:**

All four serous and six out of nine mucinous benign tumours were diploid. All eight serous and five mucinous malignant tumours were aneuploid. Nine of eleven (81.8%) serous and all three mucinous borderline tumours were aneuploid. There were highly significant differences in mean aneuploid cells percentage between serous benign (1.5%), borderline (45.6%) and malignant (74.5%) (p = 0.0001) and between mucinous benign (13.2%) and both borderline (63.7%) and malignant (68.4%) groups (p = 0.0001). There were significant differences in nuclear area between serous benign (26.191%), borderline (45.619%) and malignant (67.634 %) and a significant positive correlation between mean percentage aneuploid value and mean nuclear area in all serous and mucinous groups.

**CONCLUSION:**

We suggest that DNA ploidy and nuclear area combined, may be adjuncts to histopathology; in ovarian serous and mucinous benign, borderline and malignant neoplasms; identifying the aggressive borderline tumours.

## Introduction

Ovarian carcinoma is one of the leading causes of death in patients with gynecological malignancy all over the world, representing about 30% of all carcinomas of the female genital organs [1]. Surface epithelial carcinomas constitute about 90% of all ovarian carcinomas. Borderline ovarian tumors account for 15-20% of all ovarian epithelial tumors [2].

Typically this cancer has an insidious onset, and worse prognosis, consequently 70% of women present with disease that has spread beyond the ovary, resulting in a high mortality rate despite optimal surgery and aggressive chemotherapy [3]. Hence, the discovery of ways to diagnose ovarian cancer at an early stage and establish more effective therapies is a critical and urgent issue [4].

In Egypt, tumours of the female genital system represent 4.1% of total malignancies, ovarian cancer representing 1.37% of them. Surface epithelial tumors represent 73.33% of ovarian tumors. Serous cystadenocarcinoma represents 34.82% and mucinous cystadenocarcinoma represents 17.04% of them, referred to hospital-based cancer registry of National Cancer Institute (N.C.I) in Egypt during the years 2003-2004 [5].

Ovarian surface epithelial tumours are classified into the following histological subtypes: serous, mucinous, endometrioid, clear cell, transitional cell, squamous, mixed, and undifferentiated [6]. Usually each subtype can be classified as benign, borderline, and malignant [7]. Patients with borderline tumours have an excellent prognosis, but five-year survival rates for patients with advanced stage cancers are less than 25% [8].

Histological type of ovarian cancer is one of the major prognostic factors determining clinical outcome. Poorly differentiated tumors are characterized by high metastatic rate and aggressiveness that influence the treatment outcome [9].

Borderline tumours show some of the features of ovarian carcinomas (nuclear atypia, high mitotic activity, stratification, glandular complexity, branching and papillary fronds) but they lack stromal invasion. However, it is important to separate borderline tumours from their invasive counterparts because of their superior prognosis [10].

Although most patients with borderline tumours can be cured by surgical excision and majority of patients with borderline ovarian tumours have an excellent prognosis, apparently 15% may suffer from recurrence and die from disease [11].

The identification of patients most likely to suffer recurrence after primary surgical treatment remains an important clinical issue. Investigations into relationship between tumor recurrence and histologic subtype, cytologic atypia and invasiveness of extraovarian implants had lead to inconsistent results. This inconsistency may be partly due to qualitative parameters being difficult to reproduce [12]. DNA cytometry may supplement subjective morphologic grading by providing objective and reproducible prognostic indices [13].

Independent prognostic factors in patients with epithelial ovarian borderline tumors are DNA-ploidy, international FIGO-stage, histologic type and patient ’s age. Studies on other molecular markers have not yet uncovered a reliable prediction of biologic behaviour; however, there is hope that future studies of genetics and molecular biology of these tumors will lead to useful laboratory test [14, 15].

Histologic grading is very important for treatment decisions in ovarian cancer. All grading systems contain a significant subjective component, which could be reduced by including objective measurements into the diagnostic decision. Image analysis was used to determine nuclear area and ploidy distributions in patients with epithelial ovarian cancer. The number of nuclei with very high DNA content was found to be of prognostic importance. Image analysis thus provides additional prognostic information in epithelial ovarian cancer [16].

DNA ploidy and/or S-phase fraction have been used as biologic predictors of aggressive behaviour in a variety of solid tumours [17]. Recently, attention has focused on borderline lesions to determine if flow cytometry plays a role in separating potentially aggressive tumours from those which will pursue a more innocuous course [18, 19].

This study aims to study cytometric DNA ploidy and morphometric nuclear area in ovarian serous and mucinous; benign, borderline and invasive malignant epithelial tumours.

## Material and Methods

The material of this study consisted of 40 ovarian specimens from patients having surface epithelial ovarian serous and mucinous tumours. Material was obtained from pathology department, Kasr el Eini Hospital, Cairo University. Formalin fixed, paraffin - embedded tissue blocks were obtained from specimens. Each specimen was fixed in 10% buffered formalin and routinely processed in ascending grades of alcohol and xylene to be embedded in paraffin blocks. Two sections, 4 µm thick each, were cut from each block. One section was stained with Haematoxylin and Eosin (H&E) for routine histopathologic typing, grading and for morphometric study. The other section was stained with blue feulgen stain for DNA analysis.

The nuclear morphometry and DNA analysis were performed at the Pathology Department, National Research Center using the Leica Qwin 500 Image Analyzer (LEICA Imaging Systems Ltd, Cambridge, England).

### Nuclear Morphometric Analysis

The morphometric analysis was carried out with optical magnification of 400X on the routine haematoxylin and eosin stained slides. The nuclear area was measured in micrometers of a real- time image from the microscope that was visualized on the video monitor. 100-150 intact nuclei have been measured. These nuclei were selected randomly from representative regions in different fields.

### DNA Analysis

Touching” nuclei were “Cut” from each other, and cellular fragments or extraneous cells were erased prior to DNA measurements. Only separate, intact nuclei were measured. Distorted or overlapping nuclei and nuclear fragments were manually eliminated from measurement. The optical density of the selected nuclei in each microscopic field was then measured and automatically converted by the system into DNA content. Many fields were selected until the desired number of nuclei 100 - 150 had been measured. The results were displayed as a frequency histogram generated by plotting the DNA content versus the number of nuclei counted. The percentages of cells within each selected area were calculated and determined automatically by the system. Interpretation of DNA histograms was performed according to [20].

### Statistical analysis

Statistical analysis of the results was performed using the chi-square test and Z test with a P value set as < 0.05 to indicate significance.

## Results

Forty specimens of selected ovarian tumour cases were prospectively collected. 23 cases (57.5%) were serous and 17cases (42.5%) were mucinous tumors. Ovarian tumours included 13 cases of benign cystadenomas (4 serous & 9 mucinous), fourteen cases of borderline tumours (11 serous & 3 mucinous) and thirteen cases of cysadenocarcinomas (8 serous & 5 mucinous).

**Table 1:** Histopathological diagnosis of the studied cases

Diagnosis	No. of cases	%
Serous benign	4	10
Serous borderline	11	27.5
Serous malignant	8	20
Mucinous benign	9	22.5
Mucinous borderline	3	7.5
Mucinous malignant	5	12.5
**Total**	**40**	**100**

The age of the cases ranged between 21 and 75 years with a mean age of 48 years.

**Figure 1:**
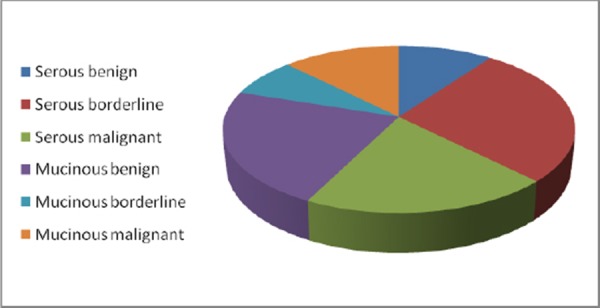
*Pie chart showing percentage of studied cases*.

All serous and six out of nine mucinous benign tumours were diploid. Only two out of eleven serous and none of the mucinous borderline tumours were diploid. All serous and mucinous malignant tumours were aneuploid.

**Figure 2:**
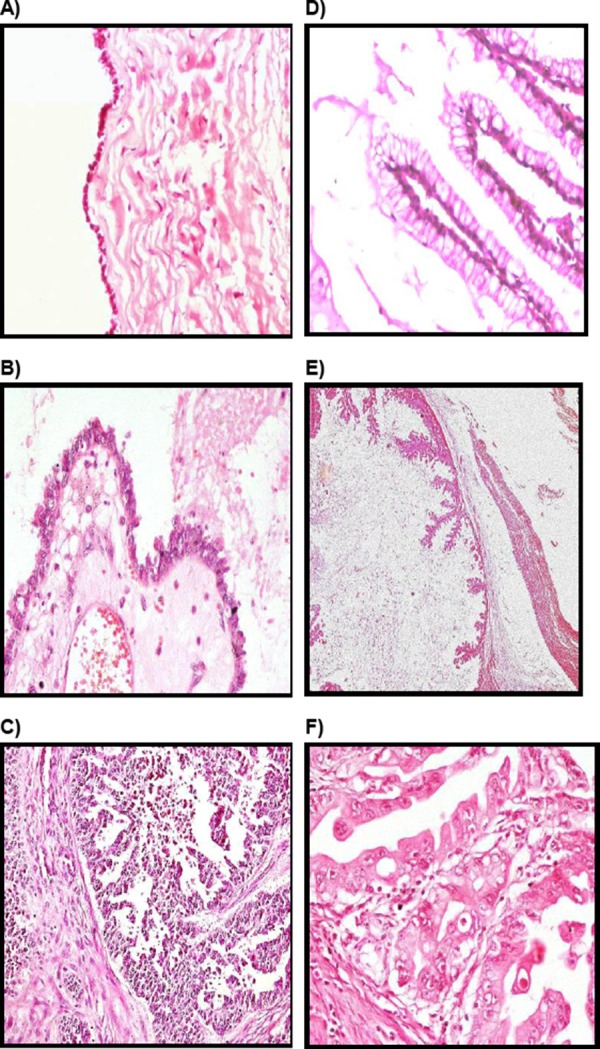
*A) Benign serous cystadenoma lined by single layer of bland-looking nonstratified cuboidal to columnar epithelial cells, (H&E, x 400). B) Borderline serous tumor showing thick papillae lined by stratified cuboidal to columnar cells with mild nuclear atypia (H&E, x 400). C) Serous cystadenocarcinoma showing areas of stromal invasion (H&E, x 200). D) Benign mucinous cystadenoma showing nonstratified columnar epithelium with abundant, pale-staining intracellular mucin and small, basal nuclei (H&E, x 400). E) Borderline mucinous tumor showing papillary infoldings lined by stratified epithelium with thin central stromal cores (H&E, x 100). F) Mucinous cystadenocarcinoma showing marked cytological atypia (H&E, x 400)*.

Highly significant differences between benign and both of borderline and malignant serous lesions were found for the percentage of diploid cells (P value < 0.05).

Highly significant differences between benign and both of borderline and malignant mucinous lesions were found for the percentage of diploid cells (P value < 0.05).

**Figure 3:**
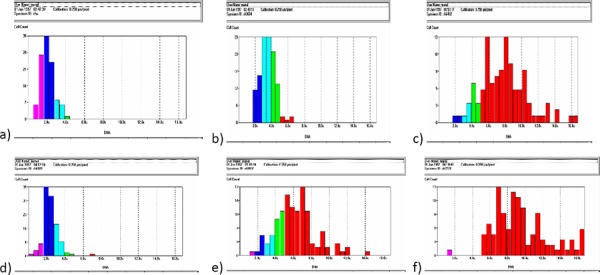
*a) Serous benign tumor with diploid histogram. b) Serous borderline tumor with diploid peak and few aneuploid cells. c) Serous malignant tumor with aneuploid histogram. d) Mucinous benign tumor with diploid histogram. e) Mucinous borderline tumor with aneuploid histogram. f) Mucinous malignant tumor with almost aneuploid cells*.

Highly significant differences between benign, borderline and malignant lesions were found for the percentage of aneuploid cells in serous tumours (>4c) (p = 0.0001).

**Table 2:** Classification of studied cases according to DNA cytometry

Diagnosis	No. of cases	Diploid cases	Tetraploid cases	Aneuploid cases
Serous benign	4 (10%)	4 (10%)	-	-
Serous borderline	11 (27.5%)	2 (5%)	-	9 (22.5%)
Serous malignant	8 (20%)	-	-	8 (20%)
Mucinous benign	9 (22.5%)	6 (15%)	3(7.5%)	-
Mucinous borderline	3 (7.5%)		-	3 (7.5%)
Mucinous malignant	5 (12.5%)		-	5 (12.5%)
**Total**	**40 (100%)**	**15 (30%)**	**3 (7.5%)**	**28 (62.5%)**

There is highly significant difference in the aneuploid value between benign and both borderline and malignant groups in mucinous tumours. Also, aneuploid value was greater in malignant than in borderline mucinous tumours, though none statistically significant.

**Table 3:** Mean diploid (2C) cell percentage in serous lesions

Diagnosis	Mean%	Std. Deviation	Std. error	Range
Benign	64.172	22.100	11.050	31.6-80.0
Borderline	6.231	7.148	2.155	0.0-18.8
Malignant	3.294	8.589	3.036	0.0-24.4

There is significant difference in the nuclear area between benign, borderline and malignant groups in serous tumours.

There is significant difference in the nuclear area between benign and malignant mucinous groups. Also, nuclear area was greater in mucinous malignant tumours than in borderline mucinous tumours; though not statistically significant and there were differences in nuclear area between borderline and both benign and malignant mucinous groups, though not statistically significant.

**Table 4:** Mean diploid (2C) cell percentage in mucinous lestions

Diagnosis	Mean%	Std. Deviation	Std. error	Range
Benign	25.509	21.903	7.301	0.8-61.4
Borderline	1.281	0.526	0.372	0.9-1.6
Malignant	2.272	2.217	0.991	0.8-5.9

The proportion of DNA-aneuploid cells in the tumours increased as the nuclear area increased. There is significant positive correlation between mean percentage aneuploid value and mean nuclear area in all serous and mucinous groups.

**Table 5:** Mean aneuploid (>4C) cell percentage in serous lesions

Diagnosis	Mean%	Std. Deviation	Std. error	Range
Benign	1.485	2.970	1.485	0.0-5.9

Borderline	45.612	33.140	9.992	4.7-92.6

Malignant	74.547	28.728	10.157	11.5-100.0

## Discussion

The hypothesis on the progression of the ovarian epithelial tumours, benign to borderline to malignant, is still controversial [21]. Tumour progression occurs mainly by dissemination through peritoneum resulting in relatively low-symptomatic disease [9]. The 5-year survival rate of women with ovarian cancer is approximately 40% and has not significantly changed over the last two decades, despite advances in treatment [22].

**Table 6:** Mean aneuploid (>4C) cell percentage in mucinous lesions

Diagnosis	Mean%	Std. Deviation	Std. error	Range
Benign	13.209	16.037	5.345	0.8-45.2
Borderline	63.678	11.629	8.223	55.4-71.9
Malignant	68.433	26.306	11.764	28.2-99.0

Morphologic studies alone cannot make a definite distinction between benignity and malignancy, nor can they identify all precancerous lesions. A prominent hallmark of most human cancer is aneuploidy, which means having an abnormal number of chromosomes in a cell; like having 45 or 47 chromosomes in a cell, when 46 is expected. Aneuploidy originates during cell division, when chromosomes do not separate efficiently between cells [23]. Aneuploidy is a result of the chromosomal instability of cancer cells and is thought to contribute to the initiation and progression of most carcinomas [15]. Aghmesheh et al, 2015 [24] stated that higher risk for aneuploidy in ovarian tumours was associated with BRCA1 mutations near the N- terminal.

**Table 7:** Mean nuclear area in serous lesions

Diagnosis	Mean%	Std. Deviation	Std. error	Range
Serous benign	26.191	4.335	2.167	23.8-32.6
Serous borderline	45.619	11.554	3.483	30.2-64.4
Serous malignant	67.634	17.288	6.112	36.0-88.6

The prognostic significance of DNA ploidy remains controversial in ovarian cancer. A number of studies on DNA aneuploidy showed that DNA aneuploidy can be of independent prognostic value [14, 25, 26]. Other studies were unable to prove the prognostic value of DNA aneuploidy [27, 28]. Our work studied DNA ploidy and nuclear area measurements in ovarian epithelial serous and mucinous tumours; benign, borderline and malignant.

**Table 8:** Mean nuclear area in mucinous lesion

Diagnosis	Mean%	Std. Deviation	Std. error	Range
Mucinous benign	35.753	7.772	2.590	25.5-46.2
Mucinous borderline	51.759	6.499	4.595	47.1-56.3
Mucinous malignant	56.982	15.672	7.008	42.3-81.9

The current study included 40 cases of ovarian surface epithelial tumours, 23 (57.5%) serous and 17 (42.5%) mucinous with benign, borderline and malignant lesions. This result agreed with that of Demirel et al, 1996 [18]; who found that serous tumours comprised the majority (74%) of their cases; the remainder were either mucinous or endometrioid tumors.

Our results showed that all serous and six out of nine (66%) of mucinous benign tumors were diploid. Diploid means having a pair of each type of chromosomes in a cell; one derived from each parent; so that the basic number of chromosomes in a somatic cell is doubled. In the normal, nearly, all somatic cells in the human body are diploid. Our study indicated that all serous benign tumours and the majority of mucinous benign tumours, proved to be diploid and thus, simulate the normal, and mostly follow an innocent course. This also agrees with Griffiths et al, 1993 [29]; who found that 55 out of their 56 cases of benign serous and mucinous ovarian cystadenomas were diploid.

**Figure 4:**
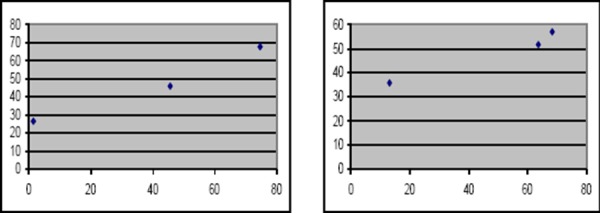
*Scatter plot 1 showing positive correlation between aneuploid value and nuclear area in serous groups (left). Scatter plot 2 showing positive correlation between aneuploid value and nuclear area in mucinous groups (right)*.

In our work, the percentage of diploid cells was significantly higher in the benign than in both borderline and malignant serous and mucinous lesions (P value < 0.05). These results came in accordance with those of Kallioniemi et al, 1988 [30]; who analyzed nuclear DNA content from ovarian tumours by flow cytometry and compared the results with the clinicopathological features of the tumours. On the basis of these clinicopathological correlations, it appeared that DNA ploidy and nuclear DNA content reflected benign behaviour of some ovarian tumours (diploid with high pecentage of diploid cells), and also reflected the malignant potential of other ovarian tumours (aneuploid); according to each case specific data, and thus complemented the routine histopathological evaluation.

Our study showed that three out of nine mucinous benign tumours were tetraploid indicating possible progression to aneuploidy. Castedo et al, 2012 [31] stated that tetraploidy has been observed in the early stages of cancer, including not only ovarian cancer, but also cervical, colorectal, esophageal, mammary and other cancers. Tetraploid cells exhibit fitness whenever there is DNA damage. This may increase their survival during tumourigenesis and after anticancer chemotherapies. Also, tetraploid cells can undergo subsequent depolyploidization. This finding of tetraploidy necessitates extensive and exhaustive gross sampling in our mucinous ovarian tumour specimens, even the benign ones; in order not to miss any hidden focus of occult or impending transition to borderline and/or malignant change.

All serous and mucinous malignant tumours in our study were aneuploid. This agrees with Pradhan et al, 2009 [32] who showed that Grades 2 and 3 serous adenocarcinomas were more often (80%) aneuploid. High grade carcinomas showing more aneuploidy than low grade ones, reveals the association between aneuploidy and high grades, and consequently the association between aneuploidy and aggressiveness. Kimmig et al, 2002 [33] revealed that DNA ploidy was valuable in predicting clinical outcome of patients with advanced cancer, its value was either similar to, or even more than that of residual disease following surgery.

Nine of eleven (81.8%) serous and all mucinous borderline tumours included in our study had an aneuploid DNA content and hence an aggressive behaviour which prompts exhaustive sampling of these tumours and close follow up of the patients; for rapid intervention. However other studies evaluated the DNA content of borderline ovarian lesions showing that (96%) of the cases were diploid and only 1 case (4%) was aneuploid [34]. Another study on borderline lesions found that (83.3%) of the cases were diploid, while (16.7%) showed aneuploid stemlines [18]. Ovarian borderline tumours included in our study, showing aneuploidy, may indicate their aggressiveness, thus addressing the issue of the necessity for rapid intervention. The variation in morphology, in this gray zone of histopathology, reflecting variation in biologic behaviour, does not only exist in borderline ovarian tumours, but also appears in colonic lesions especially adenomas where according to Gamal el Din et al., 2014 [35] mild dysplasia was seen in 26.7%, moderate dysplasia was seen in 33.3%, while marked dysplasia was observed in 40% of their colonic adenoma cases.

Our results showed, in serous lesions, a highly significant difference in the mean percentage of aneuploid cells between benign (1.5%), borderline (45.6%) and malignant (74.5%) (p = 0.0001). This agrees with Karabiowska et al, 2004 [15] who found highly significant differences between borderline and malignant lesions as regards DNA ploidy. Thus, the percentage of aneuploid cells may play a role in distinction between benign, borderline and malignant serous ovarian lesions. Also, Cohen, 1996 [36] showed that DNA aneuploidy is an independent negative prognostic factor, not only in ovarian carcinoma, but also in malignant melanoma, small cell carcinoma of the lung, esophageal, endometrial, prostatic, urinary bladder, and papillary thyroid carcinoma.

Our results showed, in mucinous lesions, highly significant differences in the mean percentage of aneuploid cells between benign (13.2%) and both borderline (63.7%) and malignant (68.4%) groups (p = 0.0001); thus agreeing with Karabiowska et al, 2004 [15]. The mean aneuploid value, in our work, was higher in malignant mucinous than in borderline mucinous neoplasms, though not statistically significant.

The frequency of DNA aneuploidy in ovarian cancer varies widely in the literature. This discrepancy in the results may be due to different methodologies (fresh versus paraffin embedded samples or flow cytometry versus photocytometry). Also, fixation process, handling of samples for DNA analysis, criteria from histograms, interpretation and intratumoural DNA heterogeneity might explain the differences in the results.

We found significant differences in the nuclear area between serous benign, borderline and malignant groups. This came in concordance with Stemberger- Papic et al, 2006 [37] who declared that there were significant differences as regards nuclear area between benign, borderline and malignant serous ovarian tumours, being highest in malignant lesions. Our study also showed significant difference in the nuclear area between mucinous benign and malignant groups, being highest in the malignant group. Differences were also found in the nuclear area between borderline and malignant mucinous groups though not statistically significant. In this context, Versa Ostojic et al, 2008 [38] found differences in the nuclear area between borderline and malignant mucinous ovarian tumours to the extent to be able to differentiate between them, nuclear area being highest in the malignant group. Also, Zeimet et al, 2011 [39] revealed prognostic relevance of nuclear area in ovarian mucinous cancer, and demonstrated correlation between nuclear area morphometric changes and early cancer genome DNA hypomethylation.

Our study showed significant positive correlation between DNA content and nuclear area in all serous and mucinous groups. These results came similar to those of Veerman et al, 2009 [19] who also showed that DNA ploidy and MNA (Mean Nuclear Area) were of significant prognostic value. Also, Lassus et al, 2011 [40] stated that DNA aneuploidy is a strong predictor of poor prognosis in serous ovarian carcinoma.

From this study, we suggest that nuclear morphometry (nuclear area measurement) and DNA cytometry (DNA ploidy studies), combined together may act as biomarkers and as adjuncts to histopathology; in ovarian surface epithelial, serous and mucinous benign, borderline and malignant neoplasms. They can segregate borderline tumours into aggressive aneuploid ones and others that will pursue a more innocent, rather indolent course. Identified patients with aggressive tumours need to be properly and early managed. This will, hopefully, lead to better therapy results. We suggest that more research work as regards ploidy-related parameters and morphometric measurements would be applied to a larger sample size of ovarian tumours. Once consistent results obtained, we suggest that DNA ploidy cytometry and nuclear area morphometry, evaluated by the image analyzer, would routinely be assessed in ovarian borderline surface epithelial serous and mucinous tumour cases.
